# Application of a rapid and accurate safe-margin volume generation method in computer-assisted bone tumor resection surgery

**DOI:** 10.1186/s13018-025-06426-3

**Published:** 2025-11-18

**Authors:** Yu Zhang, Daming Pang, Zhuoyu Li, Yang Sun, Weifeng Liu, Qing Zhang

**Affiliations:** 1 School of Astronautics, Beihang University, Beijing, 102206 China; 2 Department of Orthopaedic Oncology, Affiliated Beijing Jishuitan Hospital of Capital Medical University, Beijing, 100035 China

**Keywords:** Computer-assisted bone tumor resection surgery, Safe-margin volume generation, 3D image resampling, 3D anisotropic distance transform

## Abstract

**Introduction:**

In computer-assisted bone tumor resection, surgeons manually plan cut planes with a safe margin before surgery and follow them using navigation during osteotomy. However, manual planning is prone to error and often fails to ensure adequate margins. To address this, we propose an efficient method to rapidly and accurately generate a 3D safe-margin volume that uniformly extends the tumor by a safe margin.

**Methods:**

The study was conducted on 20 patients (9 males, 11 females) between May 2018 and October 2023. The average age was 41.75 ± 14.72 years (14–66) and the tumor types were chondrosarcoma in 5 cases, giant cell tumor in 5 cases, osteosarcoma in 2 cases, chordoma in 2 cases, Ewing sarcoma in 2 cases, spindle cell sarcoma in 1 case, osteochondroma in 1 case, chondromyxoid fibroma in 1 case and peripheral nerve sheath tumor in 1 case. The quality of the generated safe-margin volumes were assessed by visual comparison outcomes, geometric errors and maximum absolute geometric errors, time costs and clinical outcomes.

**Results:**

All 20 patients were successfully followed up, with a mean follow-up duration of 42.30 ± 18.75 months (range: 3–86 months). The generated 3D safe-margin volumes were visually closer to the ground truth compared to those from the 3D morphological dilation and anisotropic distance transform methods. The method achieved a mean geometric error of approximately 0.10 mm, significantly lower than the dilation method (up to 10.00 mm) and the anisotropic method (about 1.00 mm). The average maximum absolute geometric error was 0.1818 mm, and statistical tests confirmed significant improvements (*P*-value < 0.01). The method also exhibited favorable computational efficiency, with an average runtime of 26.82 s, substantially faster than our previous point-based method (6.29 min) and acceptable for preoperative planning.

**Conclusion:**

In this study, we developed a fast and accurate safe-margin volume generation method by combining 3D image resampling with anisotropic distance transform. This method shows strong potential in clinical practice.

## Introduction

Bone tumor should be accurately diagnosed and resected in time to prevent total loss of the affected bone and metastasis of tumor [1–9]. With the emergence of computer navigation system, it becomes possible for surgeons to accurately perform many kinds of surgeries [3, 10–14] following their pre-operatively designed surgical plans. Especially, in a computer-assisted bone tumor resection surgery, the orthopaedic surgeon performs bone osteotomies to remove the entire tumor and a continuous layer of surrounding normal tissue, following the pre-operatively planned cut planes. To effectively reduce the risk of tumor recurrence and metastasis, the thickness of the surrounding normal tissue should be no less than the safe margin specified by the Enneking staging system for bone tumors [15]. Therefore, pre-operative planning of cut planes with adequate margins (i.e., no smaller than the pre-determined safe margin) against bone tumor is critical for the patient’s post-operative outcome.

As far as we know, the orthopaedic surgeon pre-operatively places a set of cut planes outside the bone tumor by hand for guiding his or her intraoperative bone osteotomies with the computer navigation system [16–19]. To be specific, the manual cut plane planning method for a computer-assisted bone tumor resection surgery is generally conducted as follows: 1) the 3D CT image and MR image of the bone tumor segment are imported to the computer navigation system (OrthoMap 3D, Stryker, Germany), then aligned through volume or point registration; 2) the masks of the bone region are automatically segmented from the 3D CT image with an appropriate threshold; 3) the masks of the tumor region are manually annotated respectively from the 3D CT image and MR image and merged together; 4) the masks of the bone region and tumor region are respectively reconstructed to 3D models; 5) through observing the relative position of 3D bone and tumor, the orthopaedic surgeon initially places an appropriate number of cut planes around the 3D tumor, then attempts to adjust the cut planes by hand to ensure the margin of each cut plane against tumor on each image slice is no smaller than the safe margin.

However, without direct feedback of the distance from each cut plane to tumor boundary, the surgeon cannot ensure that the actual margin of each planned cut plane against tumor boundary in the 3D space is no smaller than the safe margin [2, 16–19]. If the actual margin of a cut plane against bone tumor is smaller than the pre-determined safe margin, it might increase the risk of tumor recurrence and metastasis. On the contrary, if the actual margin is greater than the safe margin, it will lead to resecting more healthy bone. Both two situations are not beneficial to the post-operative restoration of the patient. According to our investigation, Paul et al. tried to improve the positioning precision of 3D cut planes by developing a haptic device [20], in which they trained the participates on using their device to place cut planes to the target positions. However, their method is not intuitive and not easy to deploy in the real clinical scenarios. In [11], Ren et al. directly applied morphological dilation on the tumor region to generate the expanded tumor region by a safe margin. However, the spacing distances of every two adjacent voxels along each dimension of a 3D medical image are usually different, thus the expanded tumor region by this method is not accurate enough as compared to the ground truth. In our previous work [6], we proposed a method for generating a safe-margin volume that encompasses the bone tumor together with its surrounding bone tissue, using a margin determined according to the Enneking staging system for each specific resection surgery. In this method, we converted the 3D bone region and tumor region to 3D point sets, and generated the safe-margin volume by iteratively accumulating those 3D bone points locating inside the safe margin of each and every 3D tumor point. The safe-margin volume generated by this method is far more accurate than that generated by the morphological dilation method [11]. Guided by its generated safe-margin volume in the computer navigation system or surgical planning system, the orthopaedic surgeon can conveniently place a set of cut planes by hand outside bone tumor with adequate margins (i.e., no smaller than the pre-determined safe margin). However, this method was quite time-consuming compared to the morphological dilation method due to exploiting iterative search scheme, and its generated safe-margin volume was still a bit inaccurate compared to the ground truth due to the information loss during converting each voxel of the 3D image to a single 3D point. Thus, in this study, we have further developed a 3D image resampling and 3D anisotropic distance transform based safe-margin volume generation method, which is able to fast generate a 3D patient-specific safe-margin volume consisting of the bone tumor and a surrounding region within its safe margin and significantly improve the accuracy compared to the current state-of-the art methods. Overall, the proposed method is time-efficient and can fast provide the patient-specific safe-margin volume to the orthopaedic surgeon for manual planning cut planes or to the computer for automatic planning.

The main contributions of this study are threefold:

- We propose a fast and accurate safe-margin volume generation method, which is able to fast generate a patient-specific 3D safe-margin volume by extending bone tumor uniformly by a safe margin along the 3D bone region. Further, the safe-margin volume generated by our method can be used to guide the orthopaedic surgeon to perform various bone tumor surgeries.
- Our safe-margin volume generation method first utilizes the original sparse ROIs to fast estimate a coarse safe-margin volume (equivalent to the result of the method in [6]), and construct the final safe-margin volume by integrating the coarse safe-margin volume with a fine dangerous ring region reconstructed from the resampled dense ROIs. In this manner, our method can not only generate more accurate safe-margin volume than the state-of-the-art methods, but also be much faster than our previous method [6] owing to exploiting the efficient 3D anisotropic distance transform.
- We construct a safe-margin volume generation dataset, consisting of 20 sets of our previously designed surgical plans for computer-assisted tumor resection surgeries, in order to comprehensively evaluate the performance of the safe-margin volume generation method. The code implementing our method, along with the dataset used in this study, will be made available at https://github.com/uzeful/FastDRG.

## Materials and methods

### Patient population

In this study, the proposed safe-margin volume generation method has been evaluated on the previously collected 20 sets of clinical surgical plans designed by our team in Beijing Jishuitan Hospital from 2018 and October 2023. The ethical approval was granted by the Medical Ethics Committee of Beijing Jishuitan hospital. More specifically, each surgical plan consists of the aligned 3D CT image and MR image, manually annotated tumor masks respectively from the 3D CT image and MR image, and automatically segmented bone masks from the 3D CT image. The primary information of the 20 patients, including 9 males (mean age: 43.1) and 11 females (mean age: 40.6). chondrosarcoma in 5 cases, giant cell tumor in 5 cases, osteosarcoma in 2 cases, chordoma in 2 cases, Ewing sarcoma in 2 cases, spindle cell sarcoma in 1 case, osteochondroma in 1 case, chondromyxoid fibroma in 1 case and peripheral nerve sheath tumor in 1 case. According to the Musculoskeletal Tumor Society Score (MSTS) staging system, 8 tumors were stage IIB, 5 tumors were stage IB, 6 tumors were stage III and 1 tumors were stage II. Tumor locations included the pelvis (n = 15), sacrum (n = 2), tibia (n = 1), femur (n = 1), and rib (n = 1) (Table 1).

**Table 1:** Primary information of 20 patients previously undertaking computer-assisted bone tumor resection surgeries

ID	Tumor position	Tumor type	Benign/malignant	Age	Gender	Safe margin (mm)	Recurrence	Metastasis	Status	Follow-up time
1	sacrum	chordoma	malignant	60	male	15	No	Yes	Live	86
2	pubis and acetabulum	osteosarcoma	malignant	56	male	20	No	No	Dead	17
3	femur	giant cell tumor	benign	25	male	10	No	No	Live	65
4	ilium	peripheral nerve sheath tumor	malignant	39	female	20	No	No	Live	62
5	acetabulum	giant cell tumor	benign	51	female	10	No	No	Live	62
6	tibia	osteosarcoma	malignant	14	female	20	No	No	Live	61
7	rib	osteochondroma	benign	19	male	20	No	No	Live	58
8	pubis	spindle cell sarcoma	malignant	66	male	20	No	No	Live	54
9	ilium and acetabulum	chondrosarcoma	malignant	46	female	20	No	Yes	Dead	50
10	acetabulum, pubis and ischium	giant cell tumor	benign	45	female	10	No	No	Live	47
11	ilium	giant cell tumor	benign	57	female	10	No	No	Live	43
12	pubis	chondrosarcoma	malignant	45	female	20	No	No	Live	35
13	pubis	chondrosarcoma	malignant	40	female	20	No	No	Live	31
14	acetabulum	Ewing sarcoma	malignant	22	female	20	No	No	Live	31
15	ilium	chondromyxoid fibroma	benign	49	female	20	No	No	Live	28
16	pubis	chondrosarcoma	malignant	39	female	20	No	No	Live	26
17	ilium and acetabulum	chondrosarcoma	malignant	57	male	20	No	No	Live	25
18	ilium	Ewing sarcoma	malignant	19	male	20	No	No	Live	25
19	sacrum	chordoma	malignant	49	male	15	No	No	Live	20
20	ischium	giant cell tumor	benign	37	male	10	No	No	Live	20

**Fig. 1:**
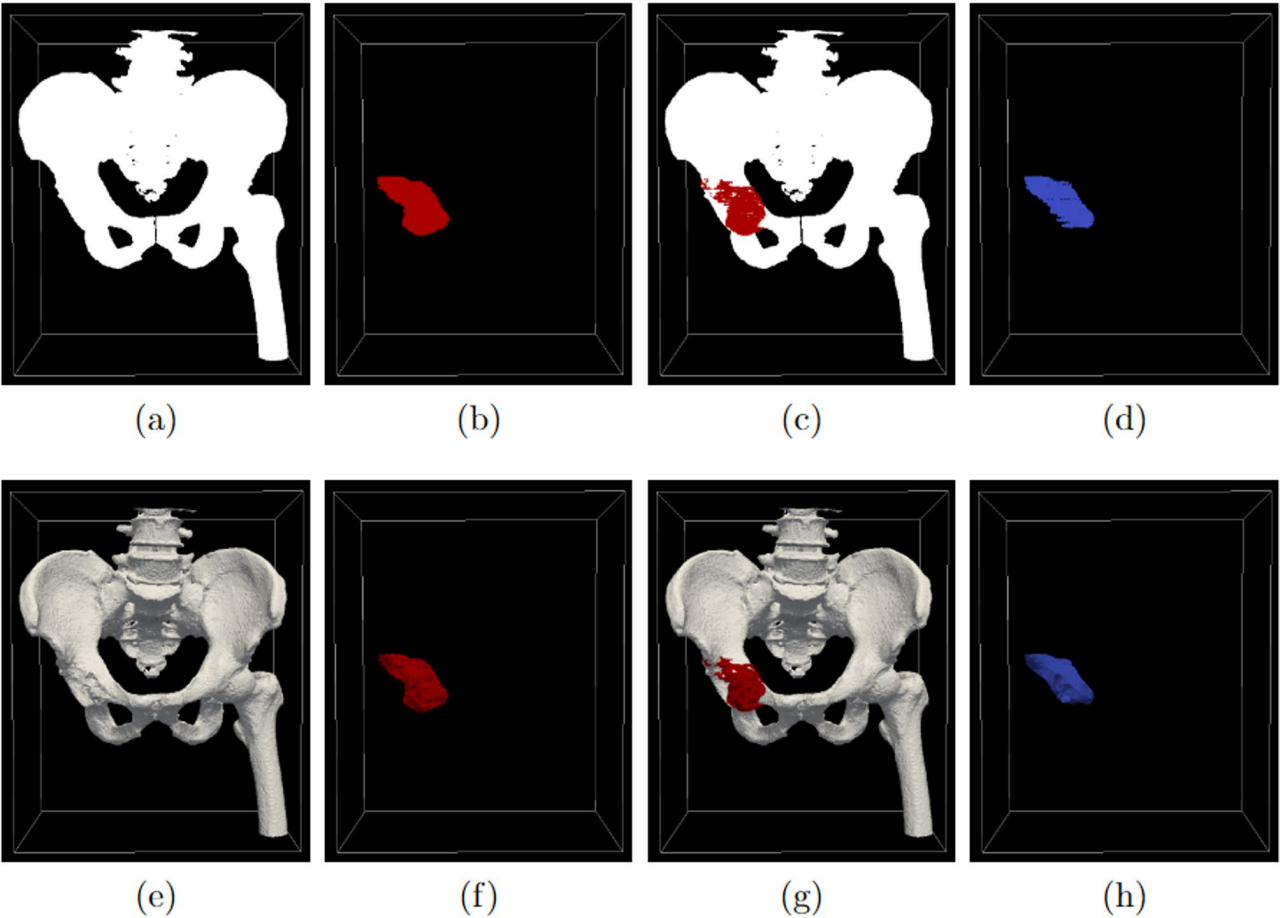
Demonstration of a set of materials for generating a patient-specific safe-margin volume. **a** and **b** respectively illustrate the 3D bone image and 3D tumor image without refinement; **c** shows the 3D tumor image overlay on the 3D bone image; **d** shows the 3D refined tumor image; **e**–**h** shows the 3D models respectively reconstructed from **a**–**d**. In the sub-figures, the units of the 3D images are in 3D voxels, and the units of the 3D models are in millimeters

In order to conveniently generate the patient-specific safe-margin volume, we have processed the original materials as follows. For each surgical plan, we first converted the automatically segmented bone masks to a 3D bone image, where the voxels located in the bone region were set to true and other voxels set to false. Given that only partial tumor region is useful for estimating the patient-specific safe-margin volume (i.e., intersected part of tumor masks and bone masks), we merged the tumor masks annotated from the 3D CT image and 3D MR image and calculated the intersected region of bone and tumor masks (i.e., where the voxels were true in the bone masks and tumor masks both), and converted the intersected region to a 3D refined tumor image. A set of materials for generating a patient-specific safe-margin volume are demonstrated in Fig. 1.

Overall, we have generated a 3D bone image (denoted by V_b_) and a 3D refined tumor image (denoted by V_t_) for each specific patient, and used them for generating the patient-specific safe-margin volumes.

### Related work

In this study, the proposed safe-margin volume generation method is essentially a 3D region expanding method, which extends the 3D bone tumor uniformly by a safe margin in the 3D space. In general, 3D region expanding can be achieved in two domains, that is, 3D image domain and 3D point cloud domain. In the 3D image domain, a 3D region can be expanded uniformly in units of voxels by the morphological dilation method [21–23] and the 3D distance transform methods [24–27]. However, in a 3D medical image, the spatial distances of every two adjacent voxels along the three dimensions are usually different, thus the morphological dilation method cannot expand the 3D region uniformly in units of millimeters in the 3D image domain. Besides the 3D morphological dilation method, 3D isotropic distance transform is also able to expand the 3D region uniformly in units of voxels in the 3D image domain. This method first calculates the 3D distance (in units of voxels) of each voxel in the 3D image to the target 3D region for expanding, then extracts the extended region as those voxels within distances less than the target margin. Similar to the 3D morphological dilation method, 3D isotropic distance transform method can only uniformly expand a 3D region in the 3D medical image of same spacing distances in all three dimensions. Further, the 3D anisotropic distance transform is developed to deal with the above problem, which considers the spacing distances in three dimensions to calculate the Euclidean distances of each voxel in the 3D image to the target 3D region for expanding. In this way, the calculated distances will be in units of millimetres rather than voxels, and the extended region generated by this method will be far more accurate.

As in the 3D point cloud domain, the 3D extended region can be obtained by greedily searching and accumulating the 3D points locating inside the scope of each 3D point in the target 3D region for expanding [6]. This method can generate the 3D region accurately, but its time cost will be increased heavily with the increase of point density. Kdtree [28] can be used to boost the efficiency of 3D point retrieval, but this method requires a large amount of memory space to store the relative positions of all the 3D points.

More specifically, in this study, we aim to expand the 3D tumor uniformly by a safe margin in the 3D space with a condition that the expanded region should be within the 3D bone. Such that resection of the entire safe-margin volume could ensure removal of the tumor region and a surrounding layer of normal tissues. As for this topic, there is only one existing method, that is, a simple but time-consuming safe-margin volume generation method [6] we previously proposed in the point cloud domain. Thus, we have introduced this method in details in the following part.

In this method, the 3D point set of the diseased bone and that of the 3D tumor are generated by converting the 3D voxels in their annotated masks to 3D points, respectively. The transformation from a 3D voxel to a 3D point can be expressed as:

1 $$ \left\{ {\begin{array}{*{20}l} {p_{x}^{i} = \left( {i - 1} \right) \times S_{x} + P_{x} } \hfill \\ {p_{y}^{j} = \left( {j - 1} \right) \times S_{y} + P_{y} } \hfill \\ {p_{z}^{k} = P_{z}^{k} } \hfill \\ \end{array} } \right., $$

where [${p}_{x}^{i}$,${p}_{y}^{j}$,${p}_{z}^{k}$] are the 3D coordinates of the voxel located at [$i$, *j*, *k*] in the 3D image V, and [${P}_{x}$,${P}_{y}$,${P}_{z}^{k}$] are the 3D coordinates of the patient’s position on the *k*th slice of V.

Afterwards, the interaction part of 3D bone and tumor, denoted by *P*_*int*_, is extracted as those tumor points locating inside the 3D bone. Generally, the partial tumor locating outside the bone region does not directly contact the patient’s bone, thus it should not lead to recurrence of bone tumor. Therefore, only the intersected part of bone and tumor should be used for generating the safe-margin volume. Otherwise, the safe-margin volume would be overestimated, and guide the surgeons to resect much more healthy bone than those healthy bone within the tumor’s safe margin.

Let us denote the point sets of 3D bone, 3D tumor and 3D safe-margin volume by *P*_*b*_, *P*_*t*_ and *P*_*d*_, respectively. Then, our previous safe-margin volume generation method can be summarized as follows: First, the tightest boundary of *P*_*b*_, denoted by *B*_*b*_, is generated by the alpha-shape method [29]. Then, the intersected part of *P*_*b*_ and *P*_*t*_, denoted by *P*_*int*_, is extracted as those points located inside *B*_*b*_ from *P*_*t*_. Finally, *P*_*d*_ can be generated by iteratively extracting from *P*_*b*_ as those bone points located within the safe margin of each and every point in *P*_*int*_, which can be mathematically expressed as:

2 $$ P_{d} = \left\{ {p_{j} \left| {dist} \right.\left( {p_{j} ,p_{k} } \right) \le d_{sm} ,\forall p_{k} \in P_{{\mathrm{int}}} ,\forall p_{j} \in P_{b} } \right\}, $$

where $dist$(${p}_{j}$, ${p}_{k}$) denotes the Euclidean distance between ${p}_{j}$ and ${p}_{k}$, and ${d}_{s}$ denotes the safe margin determined according to the stage of bone tumor.

The average time cost of this method for generating a patient-specific safe-margin volume was 6.29 min on our previously used dataset. Moreover, the precision of the generated safe-margin volume is directly related to the spacing distances of the 3D medical image, and the spacing distances along three dimensions in our collected data are often close to [0.80, 0.80, 1.00 mm]. Thus, approximating a voxel occupying a spatial space of 0.80 × 0.80 × 1.00 mm by a single 3D point is far from accurate for the precise resection of bone tumor. Further, without incorporating any computation acceleration technique, the time cost of this safe-margin volume generation method is very high because it iteratively searches the locally nearest bone points around each tumor point.

Overall, the safe-margin volumes generated by the existing methods are still not accurate enough, and the time costs of these method are relatively high. To deal with these issues, we first crop the regions of interest from the 3D bone image and tumor image to reduce the basic computational cost of our method, then develop a new 3D medical image resampling method to improve the spatial resolution of the cropped 3D images. Afterwards, we exploit the 3D anisotropic distance transform to efficiently generate a coarse safe-margin volume from the original cropped 3D images without resampling, and a fine dangerous ring region from the resampled cropped 3D images. Finally, the coarse safe-margin volume and the fine dangerous ring region are combined together as our final safe-margin volume. In this way, our method can not only improve the precision of the generated safe-margin volume compared to the single 3D anisotropic distance transform method and the 3D morphological dilation method, but also have relatively high efficiency.

## Safe-margin volume generation method

According to the descriptions in Related works, there still exist many problems in the related methods for generating the bone tumor related safe-margin volume. Therefore, in this study, we target on solving the existing key problems encountered in safe-margin volume generation, and propose a fast and accurate safe-margin volume generation method for the computer-assisted bone tumor resection surgery.

**Fig. 2:**
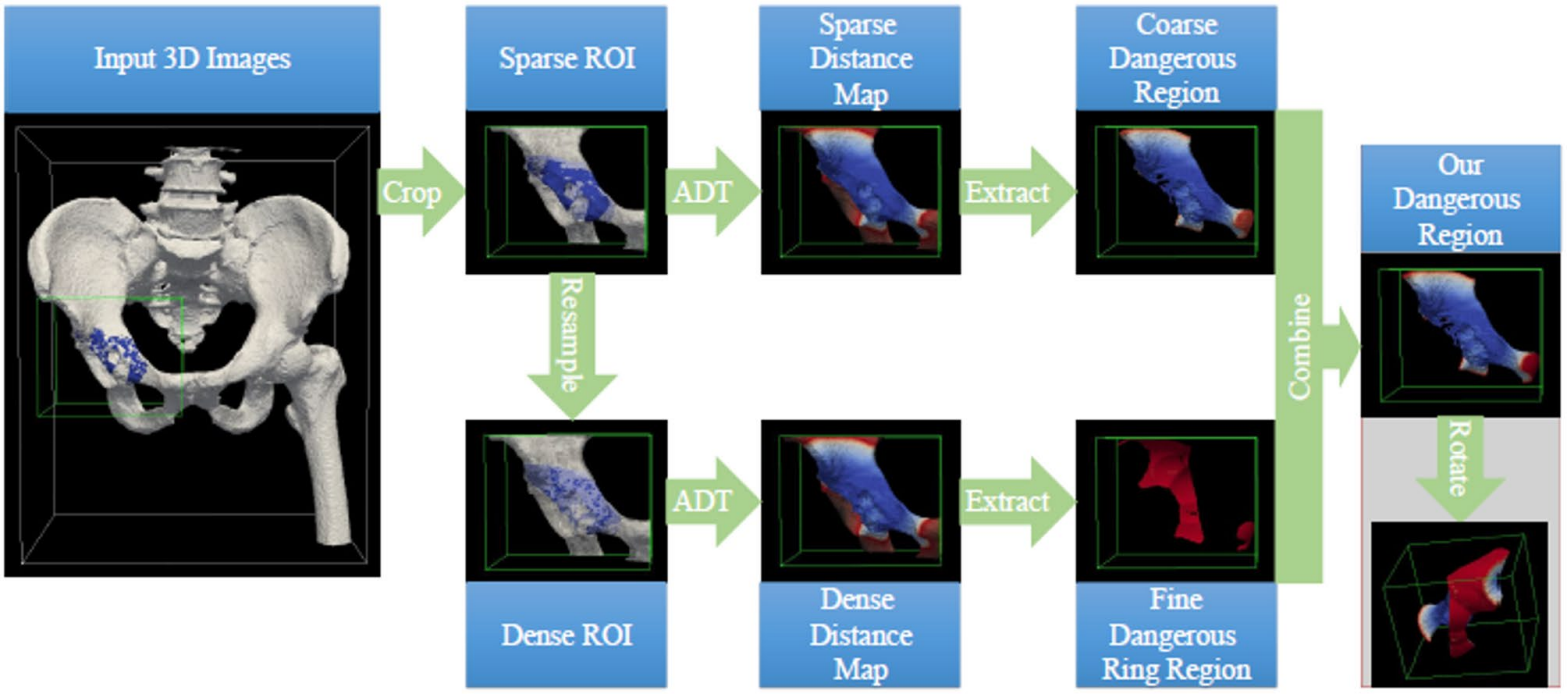
Flowchart of our proposed fast and accurate safe-margin volume generation method. In this figure, the green box indicates the bounding box of our extracted ROIs, and ADT represents the 3D anisotropic distance transform method. The surface color of the 3D models in the bottom three rows indicates the Euclidean distance from the current position to the tumor region, and the surface color is closer to red if current position is closer to the safe margin of the tumor region. Note that our method is actually performed on the 3D images, but the procedures of our method are all illustrated with the 3D models reconstructed from the corresponding 3D images for better visual effect

For each specific surgery, the regions of interest (ROI, i.e., a 3D cuboid image consisting of the tumor region and a continuous layer of surrounding normal tissue) are first cropped from the patient’s 3D bone image and 3D refined tumor image, respectively. In this manner, the time cost and required random-access memory for deploying the safe-margin volume generation method can be reduced significantly. Then, the cropped ROIs are resampled to increase their spatial resolution, so that the precision of the generated safe-margin volume could be significantly improved owing to the more precise transformation from the resampled 3D images to the dense 3D point sets. Afterwards, with the original sparse ROIs and the resampled dense ROIs, we develop a 3D anisotropic distance transform based safe-margin volume generation method for efficiently estimating the accurate but relatively sparse patient-specific safe-margin volumes. The flowchart of our proposed method is illustrated in Fig. 2, and the detailed procedures of our method are elaborated in the following subsections.

### Cropping regions of interest

Regions of interest (ROIs) are cropped from the 3D bone image *V*_*b*_ and 3D refined tumor image *V*_*t*_ according to Eqs. (3) and (4), respectively.

3 $$ V_{b}^{c} = V_{b} \left( {x_{ROI}^{\min } :x_{ROI}^{\max } ,y_{ROI}^{\min } :y_{ROI}^{\max } ,z_{ROI}^{\min } :z_{ROI}^{\max } } \right), $$

4 $$ V_{t}^{c} = V_{t} \left( {x_{ROI}^{\min } :x_{ROI}^{\max } ,y_{ROI}^{\min } :y_{ROI}^{\max } ,z_{ROI}^{\min } :z_{ROI}^{\max } } \right), $$

where B$$_{{ROI}} $$=$$ \left[ {\begin{array}{*{20}c} {x_{{ROI}}^{{\min }} ,y_{{ROI}}^{{\min }} ,z_{{ROI}}^{{\min }} } \\ {x_{{ROI}}^{{\max }} ,y_{{ROI}}^{{\max }} ,z_{{ROI}}^{{\max }} } \\ \end{array} } \right] $$denotes the bounding box of the cropped ROI, in which $$x_{ROI}^{\min } ,y_{ROI}^{\min } ,z_{ROI}^{\min }$$ and $$x_{ROI}^{\max } ,y_{ROI}^{\max } ,z_{ROI}^{\max }$$ are two extreme corners of the ROI’s bounding box. Specifically, *B*_*ROI*_, a bounding box that encloses the refined tumor region and an extra surrounding region whose thickness is a bit greater than the pre-determined safe margin *d*_*s*_, can be computed as:

5 $$ \begin{array}{*{20}c} {B_{ROI} = \left[ {\begin{array}{*{20}c} {x_{t}^{\min } ,y_{t}^{\min } ,z_{t}^{\min } } \\ {x_{t}^{\max } ,y_{t}^{\max } ,z_{t}^{\max } } \\ \end{array} } \right] + \left( {d_{s} + \xi } \right) \times \left[ {\begin{array}{*{20}c} { - 1, - 1, - 1} \\ {1,1,1} \\ \end{array} } \right]} \\ { = \left[ {\begin{array}{*{20}c} {x_{t}^{\min } - d_{s} - \xi ,y_{t}^{\min } - d_{s} - \xi ,z_{t}^{\min } - d_{s} - \xi } \\ {x_{t}^{\max } + d_{s} + \xi ,y_{t}^{\max } + d_{s} + \xi ,z_{t}^{\max } + d_{s} + \xi } \\ \end{array} } \right],} \\ \end{array} $$

where $$B_{t} = \left[ {\begin{array}{*{20}c} {x_{t}^{\min } ,y_{t}^{\min } ,z_{t}^{\min } } \\ {x_{t}^{\max } ,y_{t}^{\max } ,z_{t}^{\max } } \\ \end{array} } \right]$$ denotes the bounding box of 3D refined tumor in Vt, $x_{t}^{\min }$, $y_{t}^{\min }$ and $z_{t}^{\min }$ are the smallest *x*, *y* and *z* coordinates of the refined tumor region, respectively. $\xi$ is a positive integer used to ensure that the entire safe-margin volume could be cropped into the ROI.

### 3D image resampling

Previously in [6], we directly transformed each voxel with true value from the 3D bone image and 3D tumor image to a single 3D point. However, the spacing distances of every two adjacent 3D voxels along each dimension in our collected CT images are usually no smaller than 0.75 mm, and the solved 3D point only indicates the center of this 3D voxel. Thus, the geometrical error of the safe-margin volume generated by our previous method with respect to the ground truth would be at least 0.375 mm, i.e., half of the minimum spacing distance. In order to deal with the above problem, we have proposed an efficient 3D image resampling method to resample the cropped ROIs (i.e., ${V}_{b}^{c}$ and ${V}_{t}^{c}$), which can adjust the spacing distances of every two adjacent resampled voxels along all three dimensions to approach a desired value (e.g., 0.10 mm). Let us denote the original spacing distances along x dimension (i.e., left–right direction) and y dimension (i.e., anterior–posterior direction) respectively by *S*_*X*_ and *S*_y_, denote the target spacing distance between every two adjacent voxels along each dimension by *S*_*t*_, and denote the target resampling coefficients along *x* and *y* dimensions respectively by *C*_*x*_ and *C*_*y*_. Then we could have:

6 $$ \left\{ {\begin{array}{*{20}c} {\left( {H_{x} \times C_{x} } \right) \times S_{t} \ge H_{x} \times S_{x} } \\ {\left( {H_{y} \times C_{y} } \right) \times S_{t} \ge H_{y} \times S_{y} } \\ \end{array} } \right., $$

where $${H}_{x}$$ and $${H}_{y}$$ denote the voxel number of the current slice along $$x$$ dimension and that along $$y$$ dimension, respectively. Accordingly, $${H}_{x}\times {C}_{x}$$ and $${H}_{y}\times {C}_{y}$$ denote the voxel number of the resampled slice along $$x$$ dimension and that along $$y$$ dimension, respectively. By constraining that $${C}_{x}$$ and $${C}_{y}$$ satisfy Eq. (6), the actual spacing distance along $$x$$ dimension (denoted by $${\widehat{S}}_{x}$$ and that along $$y$$ dimension (denoted by $${\widehat{S}}_{y}$$) will be no greater than $${S}_{t}$$. More specifically, the major derivation procedures are as follows: $${\widehat{S}}_{x}$$ = $$ (H_{x} \times S_{x} ) $$$$/(H_{x} \times C_{x} ) $$$$ = S_{x} /C_{x} \le S_{t}$$$${\text{ and }}\hat{S}_{y} $$$$ = (H_{y} \times S_{y} )$$$$/(H_{y} \times C_{y} ) $$$$ = S_{y} /C_{y} \le S_{t} $$. Besides, we could get the formulation of $${C}_{x}$$ and $${C}_{y}$$ as:

7 $$\left\{\begin{array}{c}{C}_{x}\ge {S}_{x}/{S}_{t}\\ {C}_{y}\ge {S}_{y}/{S}_{t}\end{array}\right..$$

For convenient computation, we constrain all the resampling coefficients are positive odd integers in this study, and let us set ${C}_{x}=2{k}_{1}-1$ and ${C}_{y}=2{k}_{2}-1$ where ${k}_{1}$ and ${k}_{2}$ are two positive integers. Then, substituting ${C}_{x}$ and ${C}_{y}$ by (${C}_{x}=2{k}_{1}-1$) and $({C}_{y}=2{k}_{2}-1$) in Eq. (7), respectively. We could get:

8 $$\left\{\begin{array}{c}{k}_{1}\ge ({S}_{x}/{S}_{t}+1)/2\\ {k}_{2}\ge ({S}_{y}/{S}_{t}+1)/2\end{array}\right..$$

Let us set ${k}_{1}$ and ${k}_{2}$ to the minimum integers that satisfy Eq. (8), we could get:

9 $$ \left\{ {\begin{array}{*{20}c} {k_{1} = ceil\left( {\frac{{S_{x} /S_{t} + 1}}{2}} \right)} \\ {k_{2} = ceil\left( {\frac{{S_{y} /S_{t} + 1}}{2}} \right)} \\ \end{array} } \right., $$

where ceil (・) is the rounding-up function.

Further, $C_{x}$ and $C_{y}$ can be solved as:

10 $$ \left\{ {\begin{array}{*{20}c} {C_{x} = 2ceil\left( {\frac{{S_{x} /S_{t} + 1}}{2}} \right) - 1} \\ {C_{y} = 2ceil\left( {\frac{{S_{y} /S_{t} + 1}}{2}} \right) - 1} \\ \end{array} } \right. $$

Along z dimension (i.e., head-feet direction), the average spacing distance $\overline{D}$ between every two adjacent slices is first calculated as:

11 $$ \overline{D} = \left( {P_{z}^{n} - P_{z}^{1} } \right)/\left( {n - 1} \right), $$

where $n$ denotes the number of slices in the current 3D image.

Similar to Eq. (6), let us denote the target resampling coefficient for slices along z dimension by ${C}_{z}$, then we could have:

12 $$({H}_{z}\times {C}_{z})\times {S}_{t}\ge {H}_{z}\times \overline{D}$$

where ${H}_{z}$ denote the voxel number of 3D image along z dimension, i.e., the slice number of the 3D image. Accordingly, ${H}_{z}\times {C}_{z}$ denotes the voxel number of the resampled 3D image along z dimension. By constraining that ${C}_{z}$ satisfies Eq. (12), the actual spacing distance ${\widehat{S}}_{z}$ along z dimension will be no greater than ${S}_{t}$. The derivation procedures are as follows: ${\widehat{S}}_{z}=$ $$\left(\begin{array}{c}{H}_{z}\times \overline{D}\end{array}\right)/\left(\begin{array}{c}{H}_{z}\times {C}_{z}\end{array}\right)=\overline{D}/{C}_{z}\le {S}_{t}$$. Besides, we could get the formulation of ${C}_{z}$ as:

13 $${C}_{z}\ge \overline{D}/{S}_{t}.$$

As addressed above, we constrain that Cz should be a positive odd integer, i.e., ${C}_{z}=2{k}_{3}-1$ where ${k}_{3}$ is a positive integer. Then substituting ${C}_{z}$ by $2{k}_{3}-1$ in Eq. (13), we could have:

14 $${k}_{3}\ge \left(\begin{array}{c}\overline{D}/{S}_{t}+1\end{array}\right)/2.$$

Let us set ${k}_{3}$ to the minimum integer that satisfies Eq. (14), we can get:

15 $${k}_{3}=\mathrm{ceil}\left(\frac{\overline{D}/{S}_{t}+1}{2}\right).$$

Afterwards, ${C}_{z}$ can be solved as:

16 $${C}_{z}=2\mathrm{ceil}\left(\frac{\overline{D}/{S}_{t}+1}{2}\right)-1.$$

**Fig. 3:**
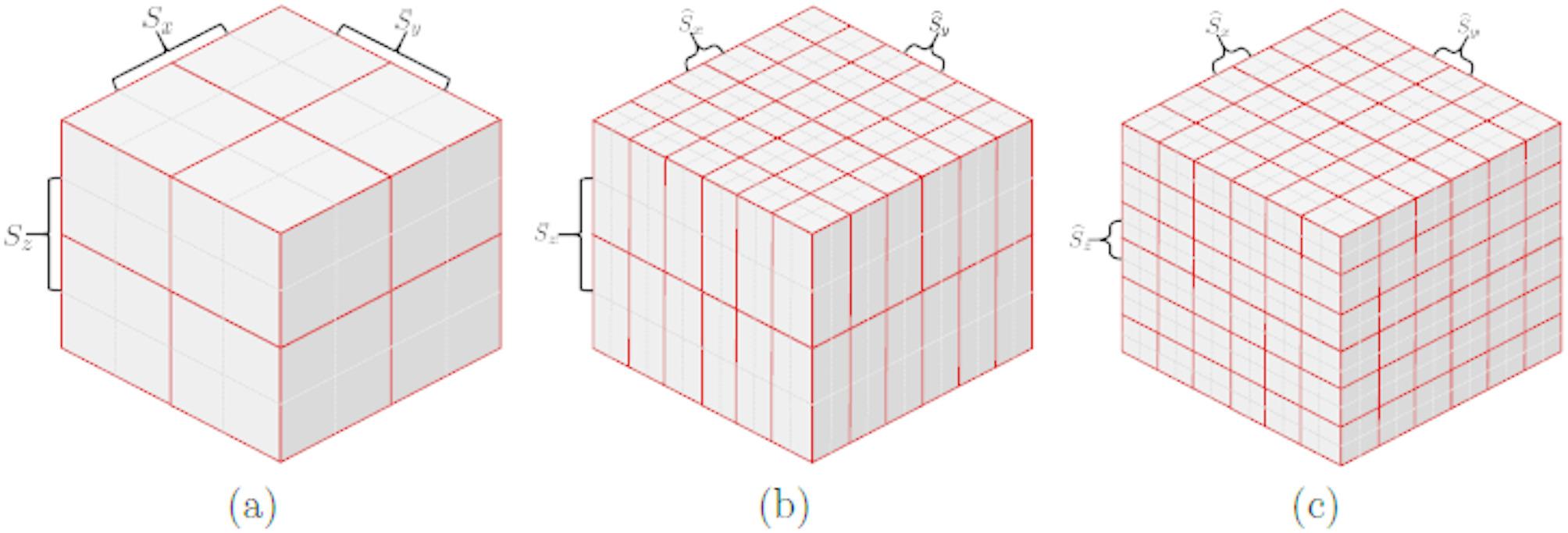
Demonstration example of our 3D image resampling method. **a** shows the original 3D image with 2 × 2 × 2 voxels; **b** shows the resampled 3D image along *x* dimension and *y* dimension, respectively; **c** shows our final resampled 3D image by further resampling **b** along *z* dimension. In this case, *C*_*x*_*, C*_*y*_ and *C*_*z*_ are all equal to 3

Assume the original spacing distances of every two adjacent voxels respectively along $x$, $y$ and $z$ dimensions are 0.75 mm, 0.75 mm and 0.80 mm respectively, and the target spacing distance *S*_*t*_ of the three dimensions is 0.10 mm. Then, we can obtain ${C}_{x}={C}_{y}={C}_{z}=9$ according to Eqs. (10) and (16). That is, in order to make the spacing distances of the resampled 3D image in three dimensions all approach 0.10 mm, the voxels in the original 3D image should be replicated 9 times along $x$ dimension, $y$ dimension and $z$ dimension, respectively. However, the resampling process for directly replicating a large 3D image 9 × 9 × 9 times would occupy a large amount of computer’s random-access memory, which makes it not applicable on most computation platforms. In order to save memory during resampling the 3D image, we first resample the slices along $x$ dimension and $y$ dimension with nearest interpolation, then further resample the slices along $z$ dimension with nearest interpolation. Given that the resampling coefficients are all constrained to be positive integers, the nearest interpolation in our method can be simply achieved by first replicating the original 3D image *C*_*x*_ times along $x$ dimension and *C*_*y*_ times along $y$ dimension and further replicating the initially resampled 3D image *C*_*z*_ times along the $z$ dimension. The resampling process can be mathematically expressed as Eq. (17) and Eq. (18), and a demonstration example is shown in Fig. 3.

17 $$ V^{\prime } = {\mathrm{repmat}}\left( {V,C_{x} ,C_{y} ,1} \right), $$

18 $$ \hat{V} = {\mathrm{repmat}}\left( {V^{\prime } ,1,1,C_{z} } \right), $$

where *V* and $\widehat{V}$ denote the original 3D image and the resampled 3D image respectively, and $V^{\prime }$ denotes the temporarily resampled 3D image. Repmat $(I,{R}_{x},{R}_{y},{R}_{z})$ denotes the repeating function, where $I$ denotes the input 3D image for resampling, ${R}_{x},{R}_{y}$ and ${R}_{z}$ are the repeating times along $x$ dimension, $y$ dimension and $z$ dimension, respectively.

After resampling, the actual spacing distances of the resampled 3D image along each dimension should be recomputed due to using the ‘ceil’ operation during computing the resampling coefficients, and the spacing distances of every two adjacent voxels along $x$ dimension, $y$ dimension and $z$ dimension can be computed respectively as:

19 $$\left\{\begin{array}{c}{\widehat{S}}_{x}=\frac{\mathrm{size}(V,1)\times {S}_{x}}{\mathrm{size}(\widehat{V},1)}\\ {\widehat{S}}_{y}=\frac{\mathrm{size}(V,2)\times {S}_{y}}{\mathrm{size}(\widehat{V},2)}\\ {\widehat{S}}_{z}=\frac{{p}_{z}^{n}-{p}_{z}^{1}}{\mathrm{size}(\widehat{V},3)-1}\end{array}\right.,$$

where size $(I,i)$ returns the voxel number of the 3D image $I$ along the $i\mathrm{th}$ dimension. ${p}_{z}^{1}$ and ${p}_{z}^{n}$ denote the $z$ coordinates of the first slice and $\mathrm{nth}$ slice of $I$, respectively.

Besides, the origin of each slice of the resampled 3D image should be updated in accordance. Before resampling the 3D image, the origin $$\left[\begin{array}{c}{P}_{x},{P}_{y},{P}_{z}^{k}\end{array}\right]$$ of each slice is located on the center of the up-left voxel of the corresponding slice. After resampling, the $x$ and $y$ coordinates of the new up-left voxel on each slice should be updated as:

20 $$\left\{\begin{array}{c}{\widehat{P}}_{x}={P}_{x}+(-{C}_{x}/2+0.5)\times {\widehat{S}}_{x}\\ {\widehat{P}}_{y}={P}_{y}+(-{C}_{y}/2+0.5)\times {\widehat{S}}_{y}\end{array}\right.,$$

As for the $z$ coordinates of the voxels on each resampled slice, we have interpolated the $z$ coordinates of every two adjacent slices in the original 3D image. First, the $z$ coordinate difference of every two adjacent slices in the original 3D image is calculated as:

21 $${D}_{z}^{i}={P}_{z}^{i+1}-{P}_{z}^{i},$$

where ${P}_{z}^{k}$ and ${P}_{z}^{k+1}$ denote the $z$ coordinates of the $\mathrm{kth}$ slice and $(k+1)\mathrm{th}$ slice in the original 3D image, respectively.

The slice number after resampling two slices will be 2*C*_*z,*_ i.e., $4\mathrm{ceil}\left(\frac{\overline{D}/{S}_{t}+1}{2}\right)-$ 2. Accordingly, the spacing distance between every two resampled slices will be $\frac{{D}_{z}^{i}}{2{C}_{z}-1}$. Therefore, the $z$ coordinate of the $j\mathrm{th}$ resampled slice between the resampled slice between the original $\mathrm{ith}$ slice and $(i+1)\mathrm{th}$ slice, i.e., the $[(i-1)\times {C}_{z}+j]\mathrm{th}$ slice of the resampled 3D image, can be computed as:

22 $${\widehat{P}}_{z}^{\widehat{k}}={P}_{z}^{i}+(j-1)\times \frac{{D}_{z}^{i}}{2{C}_{z}-1},$$

where $\widehat{k}=[(i-1)\times {C}_{z}+j]$ and $\widehat{P}{\widehat{k}}_{z}$ denotes the $z$ coordinate of the $j\mathrm{th}$ resampled slice between the original $\mathrm{ith}$ slice and $(\mathrm{i}+1)\mathrm{th}$ slice. Besides, among the resampled slices, the z coordinates of the first slice (i.e., *j* = 1) and last slice (i.e., *j* = 2*C*_*z*_) will be exactly same with those of the original two slices. As the scanning speed of the CT or MR machine might be not always the same, the spacing distance of a pair of two adjacent slices along z dimension might be slightly different to that of another pair. In this way, our 3D image resampling method could ensure the z coordinates of our resampled 3D image could be locally consistent to those of the original 3D image.

In this study, we have resampled the two sparse cropped regions of interest (i.e., ${V}_{b}^{c}$ and ${V}_{t}^{c}$) with the proposed 3D image resampling method, and obtained two dense 3D images (denoted by ${\widehat{V}}_{b}^{c}$ and${\widehat{V}}_{t}^{c}$, respectively). Next, we will exploit the above four 3D images (i.e.,${V}_{b}^{c}$,${V}_{t}^{c}$, ${\widehat{V}}_{b}^{c}$ and${\widehat{V}}_{t}^{c}$) to generate the desired safe-margin volume, and the detailed method is introduced in the next subsection.

### 3D anisotropic distance transform based safe-margin volume generation

3D anisotropic distance transform is used to calculate the 3D distance map of the foreground regions (i.e., voxels with true values), and each nonzero value in the distance map indicates the minimum Euclidean distance from the corresponding voxel to the foreground regions. Taking the 3D refined tumor region as the foreground region, we can use the 3D anisotropic distance transform to efficiently generate its 3D distance map. Specifically, the Euclidean distance of each voxel to the foreground voxels (e.g., voxels with true values in ${\widehat{V}}_{t}^{c}$) can be calculated as:

23 $$\widehat{D}\left(i,j,k\right)=\sqrt{{\widehat{S}}_{x}^{2}{d}_{x}^{2}+{\widehat{S}}_{y}^{2}{d}_{y}^{2}+{\widehat{S}}_{z}^{2}{d}_{z}^{2}},$$

where $\widehat{D}$ denotes the distance map of the tumor region in ${\widehat{V}}_{t}^{c}$, ${\widehat{S}}_{z},{\widehat{S}}_{x}$ and ${\widehat{S}}_{z}$ denote the spacing distances of every two adjacent voxels in ${\widehat{V}}_{t}^{c}$ along x and dimension, y dimension and z dimension, respectively. *d*_*x*_, *d*_*y*_ and *d*_*z*_ denote the minimum Euclidean distances (in units of voxels) between the current voxel ${\widehat{V}}_{t}^{c}\left(i,j,k\right)$ and its nearest foreground voxels in ${\widehat{V}}_{t}^{c}$ along x dimension, y dimension and z dimension, respectively.

Then, we find out the voxels within the safe margin of the tumor region, i.e., voxels whose distances are no greater than the safe margin *d*_*s*_, from the distance map$\widehat{D}$, and set the corresponding voxels to true in a new 3D image ${\widehat{V}}_{s}$ (of same size with $\widehat{D}$) and set other voxels in ${\widehat{V}}_{s}$ to false:

24 $$ \hat{V}_{s} \left( {i,j,k} \right) = \left\{ {\begin{array}{*{20}l} {{\mathrm{true}},} \hfill & {{\mathrm{if}}\;\hat{D}\left( {i,j,k} \right) \le d_{s} } \hfill \\ {{\mathrm{false}},} \hfill & {{\mathrm{Otherwise}}} \hfill \\ \end{array} } \right., $$

where ${\widehat{V}}_{s}$ denotes a 3D binary image in which only the voxels within the tumor’s safe margin are set to true.

Further, the safe-margin volume should grow along the bone region, thus the 3D image of safe-margin volume, denoted by ${\widehat{V}}_{d}$, can be generated by intersecting ${\widehat{V}}_{s}$ and ${\widehat{V}}_{b}^{c}$:

25 $$ \hat{V}_{d} \left( {i,j,k} \right) = \left\{ {\begin{array}{*{20}l} {{\mathrm{true}},} \hfill & {{\mathrm{if}}\;\hat{V}_{s} \left( {i,j,k} \right) = \hat{V}_{b}^{c} \left( {i,j,k} \right) = {\mathrm{true}}} \hfill \\ {{\mathrm{false}},} \hfill & {{\mathrm{Otherwise}}} \hfill \\ \end{array} } \right.. $$

Afterwards, the 3D point set of the safe-margin volume (denoted by ${\widehat{P}}_{d}$) can be conveniently calculated from ${\widehat{V}}_{d}$ according to Eq. (1), and the 3D safe-margin volume can be reconstructed from ${\widehat{P}}_{d}$ by the delaunay triangulation method [28].

However, the 3D point set of the dense safe-margin volume solved from the resampled ROIs (i.e., ${\widehat{V}}_{t}^{c}$ and ${\widehat{V}}_{b}^{c}$) are far more than (almost a thousand times) those of the sparse safe-margin volume solved from the original ROIs (i.e., ${V}_{t}^{c}$ and ${V}_{b}^{c}$). Thus, directly processing and storing the accurate but dense safe-margin volume solved from ${\widehat{V}}_{t}^{c}$ and ${\widehat{V}}_{b}^{c}$ will occupy a lot of random-access memory and be quite time-consuming, which make it not applicable on most computation platforms and not time-efficient for the surgeon. Therefore, we further develop a safe-margin volume generation method that can generate a safe-margin volume of high precision and run in a fast speed. The development of our finally proposed method is elaborated as below.

In general, the 3D coarse safe-margin volume (denoted by *P*_*d*_) solved from ${V}_{t}^{c}$ and ${V}_{b}^{c}$ can well represent the major structure of the safe-margin volume, and the high precision of the 3D fine safe-margin volume (denoted by ${\widehat{P}}_{d}$) solved from ${\widehat{V}}_{t}^{c}$ and ${\widehat{V}}_{b}^{c}$ is mainly owing to its accurate surface points, i.e., the points locating at the outer ring of the fine safe-margin volume. Therefore, intuitively, an accurate but relatively sparse 3D safe-margin volume (denoted by ${\widetilde{P}}_{d}$) can be generated by combining the outer ring of the 3D fine safe-margin volume (named as 3D fine dangerous ring region) and the whole 3D coarse safe-margin volume. Moreover, in this way, the point number of our generated ${\widetilde{P}}_{d}$ can be sufficiently reduced compared to that of ${\widehat{P}}_{d}$, and 3D reconstruction of ${\widetilde{P}}_{d}$ with delaunay triangulation method can be conducted very efficiently.

**Fig. 4:**
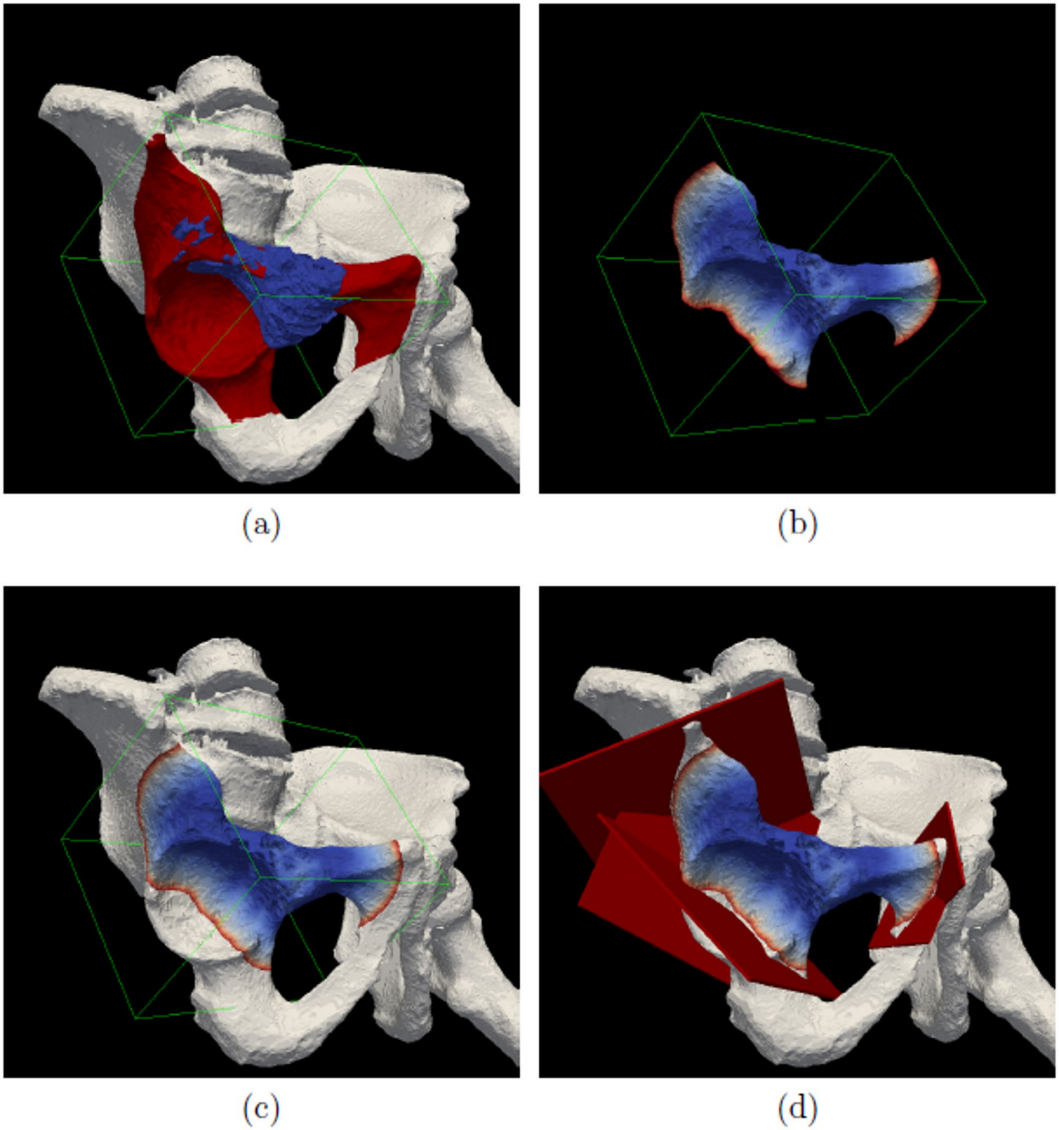
Application of our safe-margin volume generation method in surgical planning. **a** shows the 3D bone and tumor; **b** shows our solved safe-margin volume from the 3D bone image and refined tumor image; **c** shows the composition effect of the 3D bone and 3D safe-margin volume generation; **d** shows the qualified 3D cut planes planned by the method in [6] under the guidance of our generated safe-margin volume

Overall, the major procedures of our proposed safe-margin volume generation method can be summarized as follows. First, two regions of interest (i.e., ${V}_{b}^{c}$ and ${V}_{t}^{c}$) are cropped from the original 3D bone image *V*_*b*_ and 3D refined tumor image ${V}_{t}^{c}$ and respectively according to the method in Sect. ”Cropping regions of interest”. Second, ${V}_{b}^{c}$ and ${V}_{t}^{c}$ are resampled by our proposed resampling method to obtain two dense 3D images, i.e., ${\widehat{V}}_{b}^{c}$ and ${\widehat{V}}_{t}^{c}$. Third, the 3D image of the coarse safe-margin volume (i.e., *V*_*d*_) is solved from ${V}_{b}^{c}$ and ${V}_{t}^{c}$ by our proposed method at the beginning of this subsection. Then, the 3D image of the fine ring safe-margin volume (denoted by ${\widehat{V}}_{r}$) is solved from ${\widehat{V}}_{b}^{c}$ and ${\widehat{V}}_{t}^{c}$ by the same method, but there exists only one difference with the original method, i.e., we have modified the first condition $\widehat{D}\left(i,j,k\right)\le {d}_{s}$ as $\left[{d}_{s}-2\mathrm{max}\left({\widehat{S}}_{x},{\widehat{S}}_{y},{\widehat{S}}_{z}\right)\right]$ $\le \widehat{D}\left(i,j,k\right)\le {d}_{s}$ in Eq. (24). Afterwards, the true voxels in ${V}_{d}$ and ${\widehat{V}}_{r}$ are converted to 3D point sets respectively according to the transformation method in Eq. (1), which are then combined together as the final point set of our generated safe-margin volume (denoted by ${\widetilde{P}}_{d}$). Finally, the accurate but relatively sparse 3D safe-margin volume could be efficiently generated from ${\widetilde{P}}_{d}$ by the Delaunay triangulation method. An algorithm flow is shown in Algorithm 1 for better understanding of our complete safe-margin volume generation method, and an application example of our method on cut plane planning is further depicted in Fig. 4.

**Algorithm 1:**
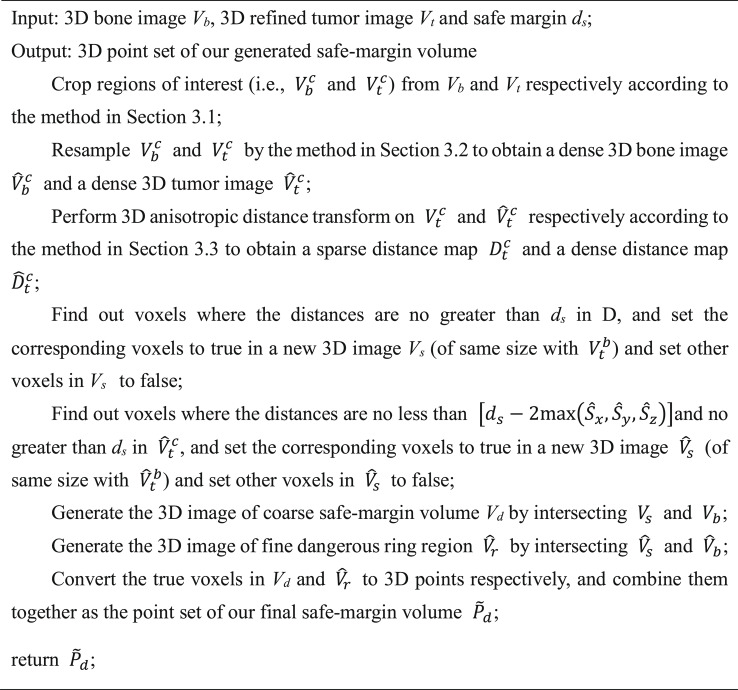
The proposed safe-margin volume generation method

All algorithmic parameters, including the target spacing (St) and replication/oddness settings, were predetermined based on prior experience and remained fixed throughout all experiments. No adjustments were made using these evaluation cases. Furthermore, the proposed method is entirely deterministic and parameter-driven, without any training or data-fitting procedure in the machine-learning sense; thus, concerns about overfitting through model training do not apply.

### Statistical analyses

To compare the geometric accuracy of the three safe-margin volume generation methods (3D morphological dilation, 3D anisotropic distance transform, and the proposed method), statistical analyses were performed on the maximum absolute geometric errors. The Anderson–Darling test was applied to assess the normality of each error distribution, and the Wilcoxon rank-sum test was used to compare the errors of the proposed method with those of each comparison method. In addition, effect sizes, expressed as mean differences with 95% confidence intervals (CIs), were calculated to provide a more comprehensive assessment of performance differences. A *P*-value < 0.05 was considered statistically significant. Survival time was defined as the interval from surgery to death or last follow-up (months). Patients alive at last follow-up were censored. Kaplan–Meier (K–M) analysis was used to estimate overall survival and corresponding 95% confidence intervals (CIs) using Greenwood’s formula. All statistical analyses were conducted in MATLAB (MathWorks Inc., Natick, MA, USA).

## Results

### Visual comparison outcomes

**Fig. 5:**
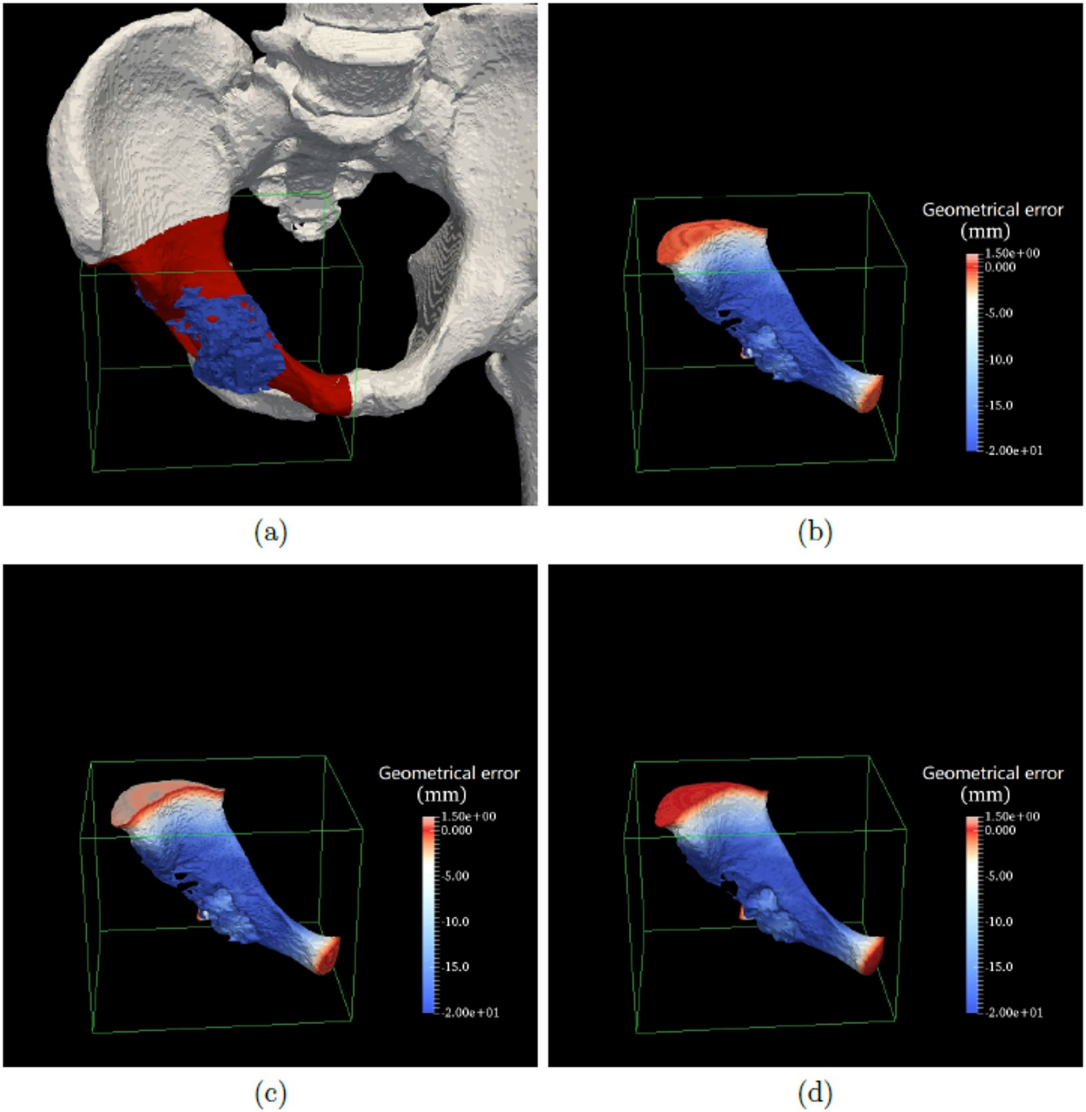
First comparison example of the three safe-margin volume generation methods. **a** shows the surgical data of the first patient in our dataset, which includes the 3D bone (in gray), 3D tumor (in blue) and our extracted ROIs (in red) in the green bounding box. **b**, **c** and **d** are the 3D safe-margin volumes generated by the 3D morphological dilation method, 3D anisotropic distance transform method and our method, respectively

**Fig. 6:**
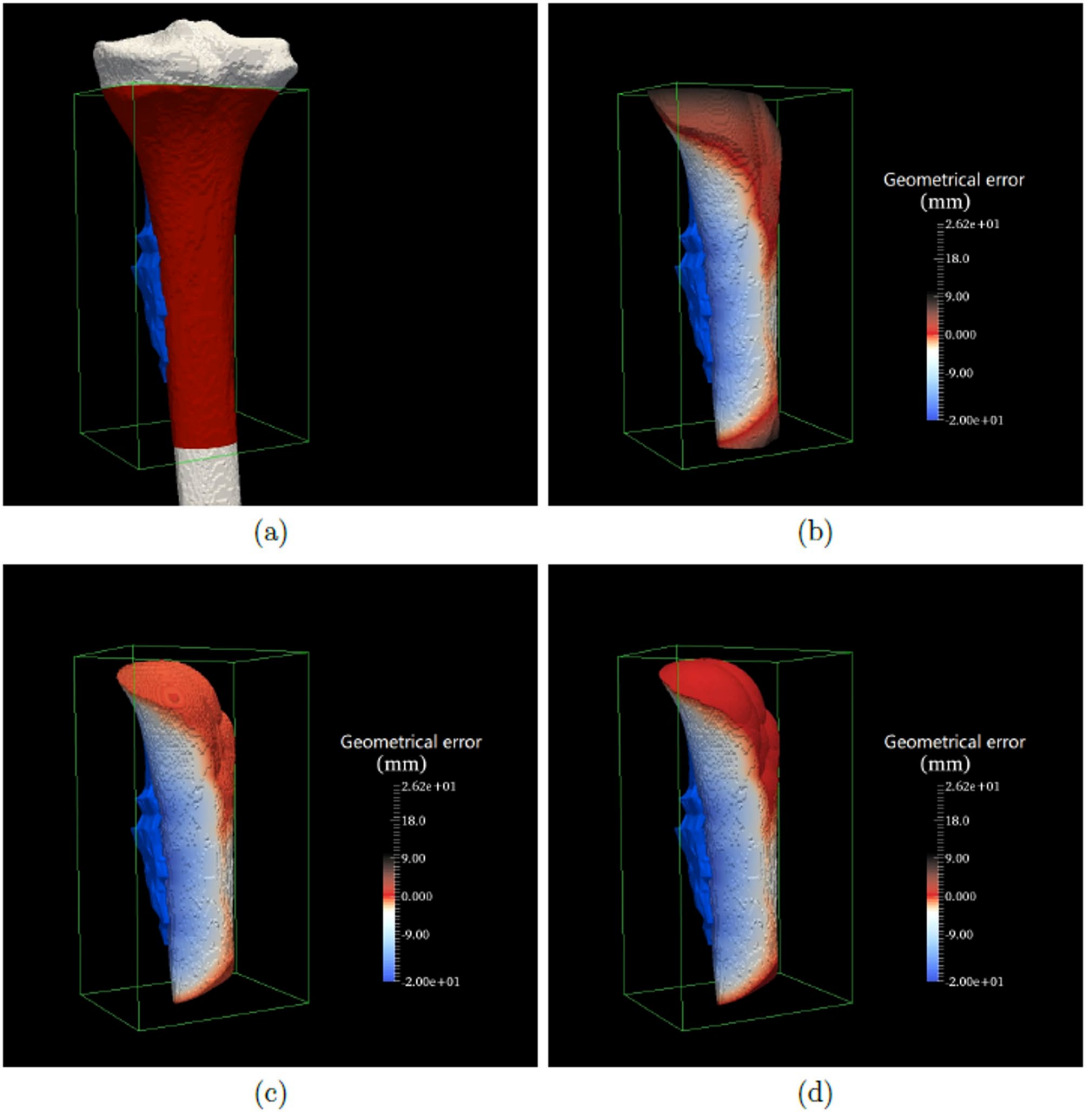
Second comparison example of the three safe-margin volume generation methods. **a** shows the surgical data of the nineteenth patient in our dataset, which includes the 3D bone (in gray), 3D tumor (in blue) and our extracted ROIs (in red) in the green bounding box. **b**, **c** and **d** are the 3D safe-margin volumes generated by the 3D morphological dilation method, 3D anisotropic distance transform method and our method, respectively

Using preoperative data from 20 bone tumor patients, 3D safe-margin volumes were generated and superimposed on the corresponding bone structures. Figures 5 and 6 present a visual comparison of the results obtained using the 3D morphological dilation method, the 3D anisotropic distance transform method, and the proposed method. As shown in the figures, the outer surfaces of the safe-margin volumes generated by our method appear significantly redder, indicating closer alignment with the ground-truth boundary. In contrast, the other two methods yielded visibly less accurate results.

Moreover, benefiting from the high-resolution resampled images, the safe-margin volumes generated by our method exhibit smoother surfaces and better visual quality. These two sets of visual comparisons clearly demonstrate the superior precision of the proposed method.

### Geometric errors and maximum absolute geometric errors

The geometric errors of the 3D safe-margin volumes generated by the 3D morphological dilation method, the 3D anisotropic distance transform method, and the proposed method were calculated, and the results are presented in Table 2. As shown, the geometric errors of the volumes generated by our method are consistently closer to zero than those of the two comparison methods. In particular, the precision of the volumes produced by our method is approximately 0.10 mm, while that of the 3D anisotropic distance transform method is about 1.00 mm. By contrast, the errors of the 3D morphological dilation method are unstable, ranging from 0.20 to 10.00 mm. These results demonstrate that our method produces more accurate safe-margin volumes than the baseline approaches.

**Table 2:** 95% CI of geometric errors of each patient-specific safe-margin volume generated by each method (Unit: Millimeters)

ID	3D Morphological dilation method	3D anisotropic distance transform method	Our method
1	[− 0.0206, 0.0107]	[− 0.7713, − 0.7602]	[− 0.0303, − 0.0300]
2	[− 0.0205, 0.0026]	[− 0.9710, − 0.9589]	[− 0.0198, − 0.0197]
3	[− 0.1943, -0.1822]	[− 0.7448, − 0.7350]	[− 0.0200, − 0.0198]
4	[− 0.2773, -0.2556]	[− 0.7946, − 0.7785]	[− 0.0180, − 0.0178]
5	[− 0.3240, -0.2994]	[− 0.9007, − 0.8756]	[− 0.0179, − 0.0175]
6	[− 0.5112, -0.4955]	[− 0.7973, − 0.7879]	[− 0.0148, − 0.0146]
7	[1.2098, 1.2817]	[− 0.8582, − 0.8453]	[− 0.0150, − 0.0147]
8	[− 0.3836, -0.3508]	[− 0.8076, − 0.7859]	[− 0.0363, − 0.0357]
9	[0.5445, 0.5742]	[− 0.8582, − 0.8732]	[− 0.0524, − 0.0520]
10	[0.0652, 0.0908]	[− 0.8590, − 0.8438]	[0.0154, 0.0151]
11	[5.9997, 6.1533]	[− 0.3903, − 0.3866]	[0.0160, 0.0159]
12	[− 0.0478, -0.0292]	[− 0.8062, − 0.7917]	[0.0137, 0.0135]
13	[− 0.7599, -0.7421]	[− 0.7898, − 0.7723]	[− 0.0150, − 0.0149]
14	[− 0.2212, -0.1851]	[− 0.7630, − 0.7284]	[− 0.0082, − 0.0078]
15	[0.5201, 0.5875]	[− 0.8662, − 0.8479]	[− 0.0173, − 0.0170]
16	[− 0.3530, -0.3249]	[− 0.8817, − 0.8607]	[− 0.0234, − 0.0230]
17	[0.0056, 0.0311]	[− 0.8979, − 0.8791]	[− 0.0190, − 0.0187]
18	[3.4743, 3.9328]	[− 1.2013, − 1.1712]	[− 0.1058, − 0.1054]
19	[9.7666, 10.0316]	[− 0.6887, − 0.6805]	[− 0.0253, − 0.0250]
20	[1.3461, 1.5361]	[− 1.1011, − 1.0780]	[− 0.1038, − 0.1032]

We further evaluated the maximum absolute geometric errors of the generated safe-margin volumes, as summarized in Table 3. The average maximum absolute geometric errors for the 3D morphological dilation method, 3D anisotropic distance transform method, and our method were 3.36 mm, 1.41 mm, and 0.18 mm, respectively. Statistical comparisons confirmed that the errors of our method were significantly smaller than those of both comparison methods.

**Table 3:** Maximum absolute geometric errors of each patient-specific safe-margin volume generated by each method (Unit: Millimeters)

ID	3D Morphological dilation method	3D anisotropic distance transform method	Our method
1	1.2445	1.1513	0.1776
2	1.5455	1.5259	0.1957
3	0.7533	1.2342	0.1835
4	0.9155	1.1702	0.1837
5	0.7331	1.6167	0.1819
6	1.3451	1.3451	0.1777
7	2.5997	1.4409	0.1777
8	0.8972	1.0667	0.1831
9	1.4217	1.2657	0.1963
10	0.8553	1.2658	0.1871
11	13.2932	0.8781	0.1766
12	0.5356	1.2321	0.1776
13	1.2072	1.2072	0.1768
14	0.6052	1.1911	0.1513
15	2.5455	1.4968	0.1818
16	1.1414	1.5379	0.1846
17	0.7435	1.5801	0.1888
18	10.0286	2.2835	0.1761
19	20.6174	1.5924	0.1778
20	8.4768	2.0593	0.2000
Average	3.5753	1.4071	0.1818

Specifically, the null hypotheses tested were that the medians of the maximum absolute errors were equal between our method and each comparator, with the alternative hypotheses being that our method achieved lower errors. The results showed highly significant improvements for our method compared with both 3D morphological dilation (*P*-value = 3.3882E—8) and 3D anisotropic distance transform (*P*-value = 3.3882E—8).

To complement the significance testing, we also report effect sizes. The mean difference in maximum absolute errors between the morphological dilation method and our method was 3.39 mm (95% CI: 0.96–5.82), while that between the 3D anisotropic distance transform and our method was 1.23 mm (95% CI: 1.08–1.37). These effect sizes confirm that our method yields substantially lower errors than both baselines, supporting the conclusions drawn from the hypothesis tests.

Since the maximum absolute geometric error of the generated safe-margin volume directly relates to the maximum surgical error in bone tumor resection, the results in Table 3 and the statistical analyses together indicate that the patient-specific safe-margin volumes generated by our method are more accurate and clinically reliable than those obtained using the 3D morphological dilation and 3D anisotropic distance transform methods.

### Time costs

The time costs of different methods for generating each patient specific safe-margin volume are counted and listed in Table 4. It can be seen from Table 4 that the average time cost of our method for generating each patient-specific safe-margin volume is about 26.82 s, which is a little longer than that of the 3D morphological dilation method (about 12.32 s) and that of the 3D anisotropic distance transform method (about 0.17 s). But the average time cost of our method (about 26.82 s) is much faster than that of our previous method (about 6.29 min), and can be neglected as compared to the time cost of the whole surgical planning stage.

**Table 4:** Time costs of different methods for generating each patient-specific dangerous region (Unit: Seconds)

ID	3D morphological dilation method	3D anisotropic distance transform method	Our method
1	3.01	0.17	26.17
2	0.50	0.26	23.83
3	0.33	0.12	10.24
4	1.17	0.10	11.44
5	0.70	0.11	9.69
6	3.89	0.15	32.28
7	10.61	0.35	66.86
8	12.49	0.18	35.81
9	6.63	0.17	37.43
10	1.31	0.14	19.25
11	108.78	0.57	56.35
12	1.67	0.10	13.13
13	6.92	0.11	18.16
14	4.43	0.27	34.64
15	0.18	0.08	8.41
16	2.18	0.12	12.52
17	0.79	0.07	11.30
18	11.71	0.10	37.19
19	62.22	0.18	22.29
20	6.96	0.11	49.31
Average	12.32	0.17	26.82

### Clinical outcomes

All 20 patients successfully underwent surgery as planned preoperatively. The mean operative time was 224.95 ± 108.83 min (range: 120–570 min), and the mean intraoperative blood loss was 1160 ± 769.03 mL (range: 100–3000 mL). Among the cases, 1 patient (5%) underwent intralesional resection, 2 patients (10%) received marginal resection, and 17 patients (85%) underwent wide resection. Pathological examination confirmed R0 resection in all patients. The average hospital stay was 24.05 ± 8.42 days (range: 12–40 days). All patients were followed up, with a mean follow-up duration of 42.30 ± 18.75 months (range: 3–86 months). Local recurrence was defined as radiologically or histologically confirmed tumor reappearance at the primary surgical site after initial complete resection. Patients were followed with X-ray and CT at 3–6 month intervals for the first 2 years, and every 6–12 months thereafter. All patients had adequate follow-up imaging to assess local control. No local recurrences were observed. Eighteen patients (90%) were alive at the last follow-up. Postoperative pulmonary metastases led to death in two patients, occurring at 17 and 50 months after surgery, respectively. The 5-year survival rate was 83.1% (95% CI: 43.0%–96.0%). One patient experienced postoperative wound non-healing, which resolved after surgical debridement.

### Complexity analysis

Finally, we report the computational platform and analyze the complexity of the proposed algorithm. All experiments were performed on a workstation equipped with an Intel Core i7-11700 K CPU @ 3.6 GHz, 128 GB RAM, and an NVIDIA TITAN Xp GPU with 12 GB VRAM. Typical memory footprints for representative ROIs are also provided.

For the time complexity analysis, let n denote the number of voxels in the cropped ROI. The distance transform operations scale linearly with the voxel count, i.e., O(n). Resampling by replication increases the voxel count in proportion to the cube of the replication factor. If the replication factor is r, the intermediate array size scales as O(r^3^n). Therefore, the overall complexity of the algorithm is approximately O(r^3^n), meaning the runtime grows linearly with ROI size and cubically with the replication factor. The effective replication factor r is determined indirectly by the chosen safe margin (ds) and target spacing (St).

## Discussion

In the surgical treatment of bone tumors, the precision of tumor resection and the control of surgical margins are fundamental determinants of therapeutic success [29]. Inadequate resection margins or positive margins significantly increase the risk of residual tumor, thereby raising the likelihood of local recurrence and adversely affecting long-term survival outcomes [13]. Conversely, in an effort to ensure negative margins, surgeons may sometimes over-resect, sacrificing more healthy tissue than necessary—particularly in anatomically critical regions near joints, nerves, or major blood vessels. Such excessive resection can lead to compromised postoperative functional recovery and diminished quality of life [1–9].

The conventional manual planning of osteotomy planes often lacks intuitive feedback regarding the spatial relationship between the tumor and the planned margins in 3D space. This limitation increases the risk of surgical inaccuracies and suboptimal outcomes [2, 16–19]. Although some studies have attempted to address this issue using haptic feedback systems or morphological dilation techniques [9], these approaches remain suboptimal. Haptic systems are generally less intuitive in clinical workflows, while morphological methods suffer from low spatial accuracy due to anisotropic voxel spacing in medical imaging [17].

To overcome these limitations in both spatial precision and computational efficiency, we proposed a safe-margin volume generation method based on 3D image resampling and anisotropic distance transform. By resampling the cropped regions of interest (ROIs) to a higher resolution and utilizing both the original and resampled images, we separately construct coarse and fine representations of the safe-margin volume, which are then fused to generate a patient-specific safe-margin volume. This approach offers high precision, rapid computation, and strong clinical adaptability.

Our experimental results provide compelling evidence for the utility of this method. In the qualitative evaluation (Figs. 5 and 6), the surface color of the generated safe-margin volumes is notably closer to red, indicating smaller geometric errors relative to the ground truth. In the quantitative evaluation, our method outperformed both the 3D morphological dilation method (which showed errors up to 10.00 mm) and the standalone anisotropic distance transform method (average error ~ 1.00 mm), achieving a mean geometric error of approximately 0.10 mm (Table 2), demonstrating excellent spatial accuracy. Additionally, our method yielded a mean maximum absolute geometric error of 0.1818 mm (Table 3), significantly lower than those of the comparison methods, with statistical significance (*P* < 0.01), underscoring its potential clinical value in minimizing intraoperative deviations.

In terms of computational efficiency, the average runtime of our method was 26.82 s. While this is slightly longer than the 0.17 s required by the non-resampled anisotropic method, it is substantially faster than our previous point-cloud-based approach, which took 6.29 min. The time cost remains well within acceptable limits for preoperative planning (Table 4), while simultaneously achieving high spatial fidelity and robustness. The clinical outcomes confirmed the feasibility of this technique and demonstrated its effectiveness in assisting surgical procedures. During the follow-up period, no local recurrence was observed in any of the patients.

According to the clinical outcomes presented in Table 1, no tumor recurrence was observed, and only two patients died due to tumor metastasis. These results suggest that the proposed method did not contribute to tumor spread and effectively supported surgeons in designing surgical plans and performing computer-assisted bone tumor resections.

Nevertheless, several limitations should be acknowledged. First, the available clinical data are limited, these observational single-arm outcomes cannot be attributed causally to the proposed algorithm and that larger comparative studies are needed to assess any clinical benefit. Second, prospective or retrospective analyses of margin adequacy, based on pathology or navigation errors, were not performed and will be addressed in future studies. Third, blinded reader evaluations, assessing planning feasibility and margin adherence with and without safe-margin volume guidance, are planned for subsequent work. Fourth, although the method fuses coarse and fine components to generate the safe-margin volume, ablation studies to isolate their individual contributions were not conducted and could be explored in the future. Fifth, the robustness of the method to segmentation errors remains untested, and future studies will assess margin violations under tumor mask perturbations. Finally, this study focuses on intraosseous resections, and extraosseous margins are not currently modeled; potential extensions to include soft-tissue margins will be investigated.

Together, these findings indicate that, while our method is computationally efficient and highly accurate for intraosseous safe-margin volume generation, further validation and methodological extensions are necessary to fully assess its clinical applicability, robustness, and generalizability to more complex surgical scenarios.

## Conclusion

In this study, we proposed a 3D image resampling scheme combined with an efficient 3D anisotropic distance transform to develop a fast and accurate method for generating safe-margin volumes, particularly for estimating regions that enclose bone tumors with appropriate safety margins. The experimental results demonstrate the method’s strong potential for clinical application. Furthermore, preliminary clinical outcomes indicate no tumor recurrence. Ongoing collection of clinical data will further validate the method’s efficacy in enhancing postoperative recovery and long-term treatment outcomes.

## Data Availability

No datasets were generated or analysed during the current study.

## References

[1] Lewis V. What’s new in musculoskeletal oncology. J Bone Joint Surg Am. 2007;89:1399–407. 17545444 10.2106/JBJS.G.00075

[2] Docquier PL, Paul L, Cartiaux O, Delloye C, Banse X. Computer-assisted resection and reconstruction of pelvic tumor sarcoma. Sarcoma. 2010;2010(1):125162. 21127723 10.1155/2010/125162 PMC2993049

[3] Wong KC, Kumta SM. Computer-assisted tumor surgery in malignant bone tumors. Clin Orthop Relat Res. 2013;471:750–61. 22948530 10.1007/s11999-012-2557-3 PMC3563803

[4] Vandergugten S, Traore SY, Cartiaux O, Lecouvet F, Galant C, Docquier PL. Mri evaluation of resection margins in bone tumour surgery. Sarcoma. 2014;2014(1):967848. 24976785 10.1155/2014/967848 PMC4058257

[5] Siegel HJ, Pressey JG. Current concepts on the surgical and medical management of osteosarcoma. Expert Rev Anticancer Ther. 2008;8:1257–69. 18699764 10.1586/14737140.8.8.1257

[6] Zhang Y, Li F, Qiu L, Xu L, Niu X, Sui Y, et al. Toward precise osteotomies: a coarse-to-fine 3d cut plane planning method for image-guided pelvis tumor resection surgery. IEEE Trans Med Imaging. 2020;39:1511–23. 31714218 10.1109/TMI.2019.2951838

[7] Hill D, Williamson T, Lai CY, Leary M, Brandt M, Choong P. Automated resection planning for bone tumor surgery. Comput Biol Med. 2021;137:104777. 34492517 10.1016/j.compbiomed.2021.104777

[8] Zhu J, Hu J, Zhu K, Ma X, Huang Z, Zhang C. Exploring the optimal reconstruction strategy for Enneking III defects in pelvis bone tumors: a finite element analysis. J Orthop Surg Res. 2025;20(1):96. 39856781 10.1186/s13018-025-05500-0 PMC11762901

[9] Guder WK, Engel NM, Hardes J, Podleska LE, Andreou D, Nottrott M, et al. Limb salvage and complication management after (sub-) total humerus resection for primary malignant bone tumors in early childhood. J Orthop Surg Res. 2025;20(1):534. 40426222 10.1186/s13018-025-05956-0 PMC12117968

[10] Marcacci M, Nofrini L, Iacono F, Di Martino A, Bignozzi S, Presti ML. A novel computer-assisted surgical technique for revision total knee arthroplasty. Comput Biol Med. 2007;37:1771–9. 17618998 10.1016/j.compbiomed.2007.05.004

[11] Ren H, Campos-Nanez E, Yaniv Z, Banovac F, Abeledo H, Hata N, et al. Treatment planning and image guidance for radiofrequency ablation of large tumors. IEEE J Biomed Health Inform. 2013;18(2):920–8. 24235279 10.1109/JBHI.2013.2287202 PMC4113118

[12] Porras AR, Paniagua B, Ensel S, Keating R, Rogers GF, Enquobahrie A, et al. Locally affine diffeomorphic surface registration and its application to surgical planning of fronto-orbital advancement. IEEE Trans Med Imaging. 2018;37:1690–700. 29969419 10.1109/TMI.2018.2816402 PMC6085886

[13] Cristoforetti A, De Stavola L, Fincato A, Mas`e M, Ravelli F, Nollo G, et al. Assessing the accuracy of computer-planned osteotomy guided by stereolithographic template: a methodological framework applied to the mandibular bone harvesting. Comput Biol Med. 2019;114:103435. 31521899 10.1016/j.compbiomed.2019.103435

[14] Hussain R, Lalande A, Guigou C, Bozorg-Grayeli A. Contribution of augmented reality to minimally invasive computer-assisted cranial base surgery. IEEE J Biomed Health Inform. 2019;24:2093–106. 31751255 10.1109/JBHI.2019.2954003

[15] Enneking WF, Spanier SS, Goodman MA. A system for the surgical staging of musculoskeletal sarcoma. Clin Orthop Relat Res. 1980;153:106–20. 7449206

[16] Wang B, Hao Y, Pu F, Jiang W, Shao Z. Computer-aided designed, three dimensional-printed hemipelvic prosthesis for peri-acetabular malignant bone tumour. Int Orthop. 2018;42:687–94. 28956108 10.1007/s00264-017-3645-5

[17] Wong KC, Kumta SM, Geel N, Demol J. One-step reconstruction with a 3d-printed, biomechanically evaluated custom implant after complex pelvic tumor resection. Comput Aided Surg. 2015;20:14–23. 26290317 10.3109/10929088.2015.1076039

[18] Cartiaux O, Docquier P-L, Paul L, Francq BG, Cornu OH, Delloye C, et al. Surgical inaccuracy of tumor resection and reconstruction within the pelvis: an experimental study. Acta Orthop. 2008;79:695–702. 18839378 10.1080/17453670810016731

[19] Masunaga T, Tsukamoto S, Honoki K, Fujii H, Kido A, Akahane M, et al. Is surgical resection of the primary site associated with longer survival in patients with metastatic chondrosarcoma at initial diagnosis? Int Orthop. 2025;49:2219–26. 40613902 10.1007/s00264-025-06600-6

[20] Paul L, Cartiaux O, Docquier P-L, Banse X. Ergonomic evaluation of 3D plane positioning using a mouse and a haptic device. Int J Med Robot Comput Assist Surg. 2009;5:435–43. 10.1002/rcs.275 19670352

[21] Van Den Boomgaard R, Van Balen R. Methods for fast morphological image transforms using bitmapped binary images. CVGIP: Graph Models Image Process. 1992;54:252–8.

[22] R. C. Gonzalez, Digital Image Processing, Pearson Education India, 2009.

[23] Haralick RM, Shapiro LG. Computer and robot vision, 1992.

[24] Felzenszwalb PF, Huttenlocher DP. Distance transforms of sampled functions. Theory Comput. 2012;8:415–28.

[25] Maurer CR, Qi R, Raghavan V. A linear time algorithm for computing exact euclidean distance transforms of binary images in arbitrary dimensions. IEEE Trans Pattern Anal Mach Intell. 2003;25:265–70.

[26] Meijster A, Roerdink JB, Hesselink WH. A general algorithm for computing distance transforms in linear time. In: Goutsias J, Vincent L, Bloomberg DS, editors. Mathematical Morphology and its applications to image and signal processing. Springer; 2002.

[27] Paglieroni DW. Distance transforms: properties and machine vision applications. CVGIP: Graph Models Image Process. 1992;54:56–74.

[28] Friedman JH, Bentley JL, Finkel RA. An algorithm for finding best matches in logarithmic expected time. ACM Trans Math Softw. 1977;3:209–26.

[29] Edelsbrunner H, Mu¨cke EP. Three-dimensional alpha shapes. ACM Trans Graph. 1994;13:43–72.

