# Correction: Application of a rapid and accurate safe-margin volume generation method in computer-assisted bone tumor resection surgery

**DOI:** 10.1186/s13018-026-07078-7

**Published:** 2026-07-14

**Authors:** Yu Zhang, Daming Pang, Zhuoyu Li, Yang Sun, Weifeng Liu, Qing Zhang

**Affiliations:** 1 School of Astronautics, Beihang University, Beijing, 102206 China; 2 Department of Orthopaedic Oncology, Affiliated Beijing Jishuitan Hospital of Capital Medical University, Beijing, 100035 China

**Correction to: Journal of Orthopaedic Surgery and Research (2025) 20:1009** 10.1186/s13018-025-06426-3

In this article [1], the corresponding author’s name Qing Zhang was incorrectly written as QingZhang.

Incorrect Name:

QingZhang

Correct Name:

Qing Zhang

The original article has been corrected.
